# Probing dynamical cortical gating of attention with concurrent TMS-EEG

**DOI:** 10.1038/s41598-020-61590-2

**Published:** 2020-03-18

**Authors:** Yuka O. Okazaki, Yuji Mizuno, Keiichi Kitajo

**Affiliations:** 1 RIKEN CBS-TOYOTA Collaboration Center, RIKEN Center for Brain Science, Wako, 351-0198 Japan; 2 Division of Neural Dynamics, Department of System Neuroscience, National Institute for Physiological Sciences, National Institutes of Natural Sciences, Okazaki, 444-8585 Japan; 3 Department of Physiological Sciences, School of Life Science, The Graduate University for Advanced Studies (SOKENDAI), Okazaki, 444-8585 Japan; 4 Research Fellow of Japan Society for the Promotion of Science (JSPS), Tokyo, 102-0083 Japan

**Keywords:** Attention, Dynamical systems

## Abstract

Attention facilitates the gating of information from the sending brain area to the receiving areas, with this being achieved by dynamical changes in effective connectivity, which refers to the directional influences between cortical areas. To probe the effective connectivity and cortical excitability modulated by covertly shifted attention, transcranial magnetic stimulation (TMS) was used to directly perturb the right retinotopic visual cortex with respect to attended and unattended locations, and the impact of this was tracked from the stimulated area to other areas by concurrent use of electroencephalography (EEG). TMS to the contralateral visual hemisphere led to a stronger evoked potential than stimulation to the ipsilateral hemisphere. Moreover, stronger beta- and gamma-band effective connectivities assessed as time-delayed phase synchronizations between stimulated areas and other areas were observed when TMS was delivered to the contralateral hemisphere. These effects were more enhanced when they preceded more prominent alpha lateralization, which is known to be associated with attentional gating. Our results indicate that attention-regulated cortical feedforward effective connectivity can be probed by TMS-EEG with direct cortical stimulation, thereby bypassing thalamic gating. These results suggest that cortical gating of the feedforward input is achieved by regulating the effective connectivity in the phase dynamics between cortical areas.

## Introduction

In our daily lives, we are bombarded with sensory inputs far beyond our information processing capacity. The covert direction of attention to specific parts of a visual scene allows processing resources to be allocated to potentially relevant stimuli, typically through the modulation of local neuronal excitability to attended and unattended sensory inputs^1–4^. Numerous studies have observed modulation of neural responses to subsequently presented stimuli at the attended or unattended location in various cortical areas of the striate^5,6^ and extrastriate^7,8^ cortices. However, given the evidence from human functional magnetic resonance imaging (fMRI) studies that top-down attention modulates activity in the lateral geniculate nucleus^9,10^, the attentional impact on cortical activities probably reflects both changes in cortical excitability and the modulation of afferent inputs through thalamic gating. Thus, it is difficult to differentiate the effect of local cortical excitability from that of afferent inputs on cortical activities. Bestmann and colleagues tested this issue by direct cortical stimulation of the visual cortex to ensure that the stimulus bypassed the thalamic gating through the retinogeniculate pathway^11^. They showed that spatial attention facilitates awareness of phosphenes induced by TMS, and their results may reflect an increase in cortical excitability in the visual cortex. Furthermore, it has been found that whether such phosphenes are perceived depends on the preceding alpha power, i.e., they are likely to be perceived at a lower alpha power^12^. Consistent with this finding, an inhibitory effect of alpha power modulation has been reported for irrelevant inputs during attention tasks. In particular, when attention is covertly directed to one visual hemifield (e.g., the left), the alpha power decreases in the contralateral hemisphere (right), but increases in the ipsilateral hemisphere (left)^13–16^. This hemispheric alpha lateralization correlates with visual detection performance^17–22^. However, it is still controversial as to whether the phosphene threshold directly reflects cortical excitability because phosphene perception is a behavioral output that depends on the participant’s subjective reports.

In addition to modulation of local cortical excitability, attention changes signal transmission, which is mediated by large-scale phase synchronizations in neural activity between task-relevant regions (we here use the term phase synchronization to denote that two interacting brain regions exhibit a constant phase difference)^23–27^. In a study using a covert visual attention paradigm, Doesburg and colleagues showed that gamma-band synchronization between the contralateral occipital electrode and other electrodes increased during attention maintenance^23,28^. Their results suggested that long-range gamma synchronization helps establish a transient network that promotes information transmission from modality-specific cortical areas to other cortical areas. On one hand, synchronizations between scalp electrodes can be a result of spurious coupling that is actually driven by a common source or contamination from volume conduction^29,30^, while on the other hand, the causal influence of one brain region on others has been assessed by measuring effective connectivity^31,32^. The most straightforward way to measure effective connectivity is to perturb a part of the brain network and observe how its impact is transmitted to other sites. In human studies, this has been achieved by combining transcranial magnetic stimulation (TMS) with EEG^31–37^. TMS-EEG has allowed demonstration of the dynamical properties of effective connectivity by showing how the propagation patterns of TMS evoked potentials could be used to differentiate between sleep and wakeful states^31^, as well as the propagation of TMS-induced transient phase resetting of ongoing oscillations from visual to motor areas^35^. In this study, we used such a perturbation approach to probe the dynamical nature of cortical excitability and effective connectivity alterations between different attention conditions. The spatiotemporal profiles of EEG responses were examined when TMS was applied to the right V1/V2 of the contralateral or ipsilateral hemisphere, depending on the attention direction.

EEG-level phase synchronization between distant brain regions may be a plausible mechanism for network communication^38,39^. If attention alters the regional composition of the network in response to the task, perturbing one of these brain regions will increase the effective connectivity between the connected regions. However, such prominent changes are not likely to be induced between disconnected regions. Furthermore, as mentioned above, alpha oscillations may play an important role in attentional function through the regulation of cortical excitability and effective connectivity. Thus, we investigated how local cortical excitability and/or inter-areal connectivity changes when the preceding alpha power is low or high, by directly perturbing the ipsilateral or contralateral V1/V2 with respect to the attention direction.

## Results

**Figure 1:**
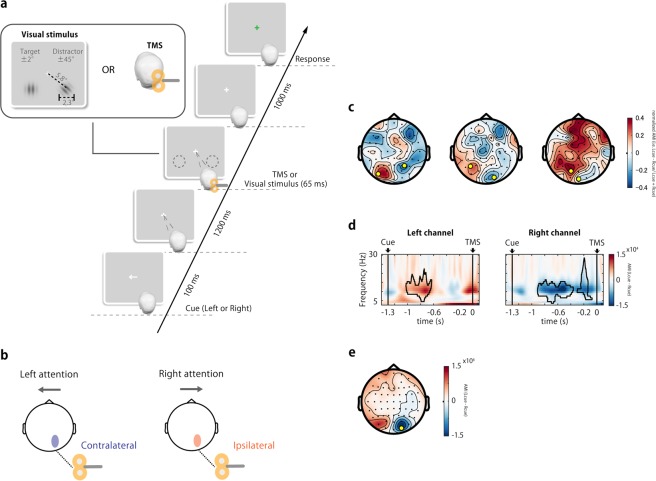
(**a**) Experimental paradigm. The trial started with a cue indicating which hemifield to attend to, and after 1.3 s, TMS was applied to the right visual cortex without presentation of a visual stimulus. To guarantee the participant’s attention to the cued hemifield, trials requiring a response to the target Gabor orientation in the cued hemifield were randomly introduced. The target Gabor stimulus (±2° oriented) in the cued side was always presented with a distractor stimulus (±45° oriented) on the opposite side. Participants were unable to predict whether a visual stimulus or TMS would be applied, so they needed to follow the cued instructions. (**b**) A schematic figure for the attention-dependent conditions. The right visual cortex where TMS was applied becomes the contralateral hemisphere in the left attention trials, while it becomes the ipsilateral hemisphere in the right attention trials. (**c**) Topography is shown as a distribution of the AMI (normalized by sum of alpha power in left and right cue) over all electrodes from three representative participants. The electrodes with the largest AMI (indicated by yellow dots) were selected for the subsequent analysis. (**d**) Grand averaged time-frequency representations of the AMI (left cue − right cue) in the left and right electrode. Alpha modulations enclosed by black lines show statistically significant clusters in the comparison of left and right cue conditions. (**e**) Grand averaged topographic AMI (8–12 Hz) for the time range from −0.5 to 0 s. The yellow dot indicates the electrode position near the TMS coil.

Participants were given TMS (real or sham) on the right V1/V2 or were presented with visual stimuli while attention was directed to either the left or right visual field according to a direction cue (Fig. 1a). In other words, TMS was applied to the visual cortex of the ipsilateral or contralateral hemisphere with respect to the attention direction (Fig. 1b). Hereinafter, all analyses were performed on data segments extracted from 3.5 seconds before to 1.5 seconds after TMS onset, with the TMS onset located at 0 sec on the time axis of figures. First, the alpha lateralization index (ALI), which is the alpha power difference between the left and right visual hemispheres, was evaluated on a trial-by-trial basis (see section *Attentional alpha power modulation* in Materials and methods). Next, to investigate the effects of attentional alpha power modulation on cortical excitability and effective connectivity probed by direct TMS perturbation, we analyzed the initial peak of the TMS evoked potential (TEP) and the time-frequency evolution of the time-delayed phase locking value (tdPLV) in high ALI and low ALI trials. The tdPLV was obtained by taking the average vector of the phase angle differences between electrodes, especially the phase differences between electrode O2 at TMS onset and other electrodes at a given lag time (see section *Inter-area phase synchronization* in Materials and methods).

### Pre-stimulus alpha power modulation by attention

To determine the left and right hemispherical electrodes that are most sensitive to attentional alpha modulation in each participant, we calculated the alpha modulation index (AMI) defined by the difference in alpha power between the left and right-cued conditions at each electrode. This means that the AMI provides the opposite sign for the left and right electrodes (Fig. 1c,e for individual and grand averaged topography). We selected the most positive and most negative electrodes among the parieto-occipital electrodes of the left and right hemispheres, respectively (as illustrated by the yellow dots in Fig. 1c).

The time frequency representation (TFR) of the power from individually selected electrodes was contrasted between left and right cue trials and averaged over participants (Fig. 1d). The grand averaged TFR of the power indicated that alpha power was continuously modulated in the interval between cue and TMS, approximately 1 s prior to the stimulus onset, and corresponding with the direction of attention. This result is evidence that the participants were engaged in the attentional task directing their attention toward the visual hemifield of the cue side, even in those TMS trials without visual stimuli, although the behavioral performance was relatively low (correct rate mean ± sd: 0.65 ± 0.11).

We also confirmed the statistical significance of alpha-power modulation by a cluster-based permutation test based on the clustering of adjacent time and frequency points on TFR between left and right attention conditions. Alpha power was significantly modulated in both hemispheres before stimulation. However, significant clusters appeared only in the first half of the attention maintenance interval (between the cue and TMS) in the left electrode, while significant clusters in the right electrode corresponding to the TMS site persisted relatively longer (Fig. 1d). These results suggest that alpha-power modulation at the right electrode was constant and persisted across participants, whereas modulation at the left electrode could be intermittent or fragmented across participants.

Topographic representation of the grand averaged AMI just before stimulation, e.g. from −0.5 to 0 s, indicates that the alpha power was clearly lateralized in the occipito-parietal electrodes, i.e., the alpha power in the ipsilateral hemisphere to the attention direction increased, and alpha power in the contralateral hemisphere decreased (Fig. 1e). Note that the TMS target (around O2) coincides with the region where alpha power was strongly modulated.

### Alpha lateralization-dependent early TMS evoked potential

**Figure 2:**
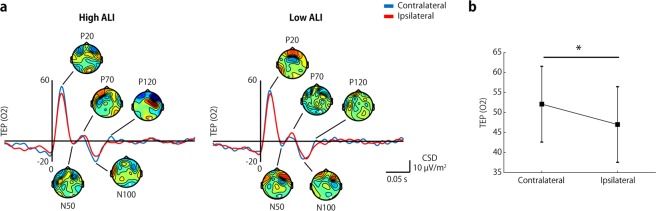
Attentional modulation in TMS evoked potentials. (**a**) TEPs from O2 at the site of stimulation for the high-ALI and low-ALI trials. Topographic differences between the left-cued and right-cued trials are shown for each TEP component. (**b**) The first response to TMS, i.e., P20, was significantly larger in the contralateral (left-cued) condition than in the ipsilateral (right-cued) condition.

The TEP was calculated to examine the response to the TMS applied to the target area, with the alpha power just before stimulation being strongly modulated by attention. After subtracting the TEP from the sham condition, we identified several TEP components from the O2 electrodes (near to the stimulation site), including the P20, N50, P70, N100, and P120 components, in both high-ALI and low-ALI trials (Fig. 2a). Cortical excitability influenced by the alpha oscillations should be reflected in an immediate response to the TMS perturbation, e.g., the P20. In high-ALI trials (left panel), the topographical map contrasting the left-cued and right-cued trials indicated that P20 showed a maximal difference at approximately the stimulation site, while the maximum differences in low-ALI trials were sparsely distributed. We quantified the effect of pre-stimulus alpha power on the P20 using a two-way ANOVA with the factors ALI type (high-ALI, low-ALI) and attention-hemisphere (ipsilateral, contralateral; Fig. 2b). This showed a main effect for attention-hemisphere (*F*(1, 17) = 8.756, *p* = 0.009), indicating that the P20 in the contralateral condition was larger than in the ipsilateral condition, regardless of the amount of alpha lateralization. There was no significant main effect for ALI type (*F*(1, 17) = 1.099, *p* = 0.309) or the interaction between factors (*F*(1, 17) = 0.587, *p* = 0.454). These results indicate that the contralateral hemisphere to the attention direction was in a highly excitable state, but that this was not likely to depend on the degree of alpha power in that hemisphere.

### Alpha lateralization-dependent effective connectivity

**Figure 3:**
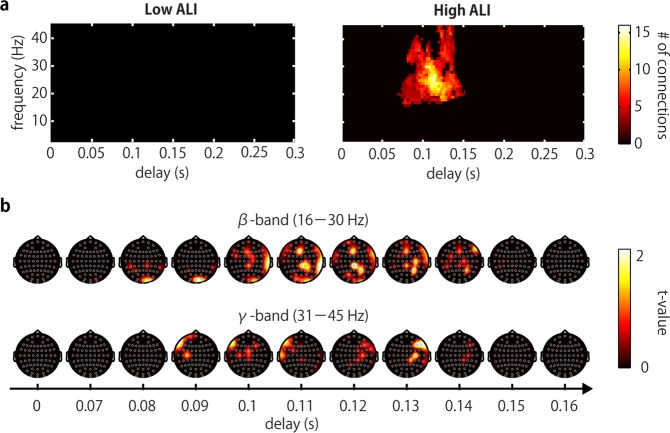
Attentional modulation in effective connectivity from V1/V2 to other areas. (**a**) A time-frequency profile of the number of significant tdPLVs in the comparison between the contralateral and ipsilateral conditions indicates that large areas are synchronized in the beta and gamma bands. This significant difference was observed only in high-ALI trials (right panel). (**b**) A spatiotemporal profile of the significant difference between contralateral and ipsilateral sides in high-ALI trials shows that TMS perturbation on the task-relevant area has strong impacts on other cortical areas.

To probe how effective connectivity varies with attention, we assessed the time-delayed phase locking value (tdPLV) to estimate the effective connectivity as a directional phase coupling between the sending and receiving areas. More specifically, tdPLV evaluates the consistency of the phase difference between electrode O2 at TMS onset and other electrodes with a given lag time (see section *Inter-area phase synchronization* in Materials and methods). Thus, the tdPLV makes it possible to know when and where the perturbed phase dynamics at one region affect other regions in the brain network. After subtracting the tdPLVs from the sham conditions, the cluster-based permutation test on tdPLVs between contralateral and ipsilateral conditions was applied to each of the low-ALI and high-ALI trials, and provided significant clusters across delay times, frequencies, and electrodes. To quantify the time-frequency characteristics of the significant clusters, we examined the number of significant electrode pairs over time and frequency range (Fig. 3a). Significant different connections were observed only in the high-ALI trial where the alpha power of the stimulation region was low under the contralateral condition and high under the ipsilateral condition (Fig. 3a right panel). There were no significant differences in the low-ALI trials (Fig. 3a left panel). In the beta and low gamma band with a peak at 25 Hz, the tdPLV of the contralateral condition was found to be significantly stronger at more electrode pairs than in the ipsilateral condition (*p* < 0.05). The significant difference started at about 70 ms, peaked at 114 ms, and ended at about 150 ms. These results show that the TMS perturbation to the contralateral hemisphere more strongly and widely affected other regions than perturbation to the ipsilateral hemisphere.

Next, we investigated the spatial extent of this difference in the beta (16–30 Hz) and gamma bands (31–45 Hz). Figure 3b shows a topographical map of the mean significant *t*-values identified by the cluster-based permutation test. For the difference between the contralateral and ipsilateral conditions in the beta-band tdPLV, the effect of TMS perturbation on the contralateral hemisphere was confined to the temporal-parietal region until about 100 ms, and then to the frontal region. For the gamma-band tdPLV, the difference was first observed in the frontal region contralateral to the stimulated site, and then moved to the ipsilateral frontal region. These results suggest that networks between short-distance areas are established with beta-band oscillations, and that the chained response was triggered by a local stimulation, whereas long distance networks may be established with gamma-band oscillations.

## Discussion

### Probing cortical excitability

It is generally agreed that directing attention toward a specific location in the visual field facilitates perception and retinotopic neural responses to subsequently presented target stimuli at the attended location^6,40^. As mentioned earlier, a prior study with direct stimulation of the neocortex suggests that these changes reflect not only attentional thalamic gating but also the regulation of cortical excitability^11^. The study showed that spatial attention facilitates awareness of phosphenes induced by TMS, although there is still controversy over the relationship between initial sensory responses and behavioral perception.

In an analogous spatial attention paradigm, we observed neural responses to TMS in early visual cortex, instead of phosphene perception. In the current study, the direct neural responses at the very early latency, i.e., P20, were modulated by the direction of attention, but were not related to alpha power modulation preceding the neural responses. These findings are compatible with a report by Herring and colleagues^41^, although in their study, the earliest attentional modulation occurred in the N40 TEP component, rather than in P20. N40 may reflect inhibitory feedback in response to TMS-induced cortical excitation, rather than the initial excitation itself^42^. A plausible explanation for the difference in timing of the attentional effect on the early TEP component may be partly related to the stimulated area. We confined the TMS target area to the right V1/V2, while Herring *et al*. defined a target area to ensure retinotopic phosphene perception. Given that it has been reported that stimulation sites generating retinotopic phosphenes are situated at V2 and V3^43^, the TEP components observed in Herring’s study might be a direct response from the extrastriate cortex, rather than the striate cortex. Such a hierarchical difference may lead to a difference in timing, and may also be related to the lack of contribution of alpha-power modulation to TEP in the present study. As the seminal study of Romei *et al*. clearly showed that whether a TMS-induced phosphene is perceived or not depends on the preceding alpha power^12^, it is plausible that the fluctuation of alpha power reflects cortical excitability. However, phosphene perception is a behavioral output that depends on the participant’s subjective report, and its awareness may require feedback signals from higher cortical areas to V1. When feedback signals were disrupted by TMS^44,45^ or damage^46,47^, awareness of a visual stimulus was impaired. In addition, reductions in feedback connectivity in an unconscious state are empirically and theoretically supported^48–52^. Although the visual experience varies with alpha oscillations, as reported by Romei *et al*.^12^, the evidence from directional connectivity raises the possibility that alpha oscillations are involved in the feedforward and feedback loop, rather than local excitability. In summary, the current results provide evidence that the top-down influences of attention on the excitability of the early visual cortices can be regulated in parallel with thalamic gating. However, the contribution of alpha oscillations to local excitability was limited.

### Probing cortical effective connectivity

The function of attention is not only to change local neural excitability, it must also facilitate dynamical interactions between different areas, depending on the task at hand. This could be achieved by flexible control of effective connectivity mediated by phase synchronization of neural oscillations in the regions sending and receiving information. Doesburg and colleagues showed that contralateral early visual cortex and other regions were coupled with gamma-band phase synchronization during covertly sustained attention^28^. To demonstrate that such phase synchronization reflects the directional network for signal transmission modulated by attention, we locally perturbed either contralateral or ipsilateral visual cortex during covert attention. In direct perturbation approaches, effective connectivity has been evaluated by measuring the propagation of evoked activity^31,33^. Although the validity of the tdPLV used here to investigate the effective connectivity is comparable to that of the techniques used in these previously mentioned studies, the tdPLV with TMS perturbation is more focused on directional interactions. When waves originating in the sending area reach the receiving area, the phase difference between these areas becomes constant at that time; that is, it becomes synchronized, but with a certain delay time. Moreover, the advantage of tdPLV is that it evaluates the phase relationship between different times, thus eliminating the effects of volume conduction, which is an instantaneous phenomenon. We found that the waves in the right early visual area propagated widely and reached to other cortical areas more in the contralateral condition than in the ipsilateral condition. This difference became significant relatively slowly, after about 70 ms. In other words, the connectivity between the contralateral and ipsilateral visual hemispheres and other regions is comparable in the early stages of visual processing, and connectivity with the contralateral hemisphere becomes dominant in the later stages. These results suggest that cortical gating of feedforward inputs may be achieved by enhancing the contralateral connections without completely disconnecting the ipsilateral connections. Importantly, successful gating depended on the degree of alpha lateralization caused by attention. Given the evidence that there was no significant effect of the degree of alpha power on the initial response of TEP, i.e., P20, the tdPLV results do not reflect the amplified activity in V1/V2. Furthermore, alpha oscillations may be more strongly involved in modulation of long-distance connectivity, rather than modulation of local excitability. Although the mechanism behind the alpha contribution and its source is still unknown, the key role in generating the alpha oscillations may be played by inhibitory GABAergic neurons^53–55^. In a model to test the cortical gating mechanism to TMS evoked activity during slow wave sleep, increased GABA release from local inhibitory neurons in the cortex was effective in reducing the propagation of evoked activity^56^. Thus, the increased effective connectivity in the present study may be mediated by GABAergic inhibitory feedback in the neocortex or thalamus. We recommend that studies employing magnetic resonance spectroscopy should be performed to reveal the roles of GABA release.

### Effect of the auditory artifact from the TMS click sound

In general, a limitation of TMS research is that the TMS pulse is accompanied by a click sound of about 100 to 120 dB^57^, as well as the introduction of several electromagnetic and/or muscle artifacts. The click sound contaminates part of the TEP with an auditory evoked potential (AEP)^58–60^. We used white noise through air tubes attached to perforated earplugs to mask the click sound, and placed a thin layer of foam between the TMS coil and the EEG cap to attenuate bone conduction of sound^31,60–62^. In addition, we arranged the electrode leads to minimize electromagnetic artifacts during the experiment^63^, and attenuated any such artifacts using offline ICA analysis^41,64^. If phase shifts are caused by an AEP and/or TMS artifacts, we should acknowledge that the tdPLV may spuriously increase, but this would not explain our results. Because the effects of these artifacts should be identical across different attention conditions and we always made comparisons between conditions with sham subtraction, we believe that the modulated tdPLV is associated with dynamical gating of cortical information processing. Nevertheless, further developments in more realistic sham stimulation^65–68^ and electrode referencing methods^69^ are awaited to allow the TMS-EEG community to maximize the direct effects of TMS on cortical responses.

### Future perspectives

The direct perturbation approach provided evidence that top-down attention coordinates cortical excitability and feedforward effective connectivity, bypassing thalamic gating. In addition, online observation of perturbation effects could characterize flexible changes in functional connectivity for feedback (top-down) control, as well as feedforward (bottom-up) processing. In voluntarily orienting attention to an object, the ventral frontal cortex in the right hemisphere is generally considered dominant, as the symptoms of spatial neglect are more often observed after a stroke in the right hemisphere than in the left hemisphere^70–72^. It would therefore be useful to further investigate the hemispheric asymmetry involved in the orienting of attention by stimulating these control regions. In this context, to generalize the current findings, we recommend stimulating the left visual cortex employing the same protocol.

## Materials and methods

### Participants

Twenty-two healthy right-handed participants (7 female and 15 male, mean age: 24.9 ± 5.7 [SD]) gave informed written consent for their participation in this study. The ethical committee of the RIKEN Center for Brain Science approved this TMS-EEG study, and the study was conducted in accordance with the Declaration of Helsinki.

### Stimulus and task

The TMS-EEG experiment consisted of six runs (three runs for real TMS, and three runs for sham TMS), each containing 96 trials, i.e., a total of 288 trials (=96 trials × 3 runs). Participants were seated 100 cm from a gamma corrected LCD monitor (BenQ XL2420, 100-Hz refresh rate) and performed a cued spatial attention task during both the real- and sham-TMS runs, which were performed in random order (Fig. 1a). A trial began with an arrow cue (0.1 s) indicating the hemifield to which the participant should attend, followed by an anticipatory interval of 1.2 s. Subsequently, either the visual stimuli were presented on both visual hemifields or TMS was applied to the right V1/V2 region (see EEG recordings and TMS), with each condition being randomly allocated to 144 trials each. Note that no visual stimuli were presented during TMS to avoid contaminating the neural activity with visual input. This allowed us to probe the current brain state with TMS. In the visual stimulation trials, a target Gabor grating (standard deviation of the Gaussian envelope, 0.18; spatial frequency, 2.5 cycles per degree [cpd]; contrast, 50%; orientation, ±2°) was presented in the cued hemifield together with a distractor Gabor grating (orientation, ±45°) in the other hemifield. These grating stimuli were presented for 0.05 s, followed by a 0.05 s bilateral backward mask stimulus (radial Gabor grating with the same property as the target). Then, after 1 s, participants were required to indicate whether the target stimulus was tilted to the right or left by pressing the arrow keys with the index or middle finger of their dominant right hand for the left or right orientation, respectively, with a response interval of 2 s being allowed for this, during which the color of a fixation cross changed from white to green. In the TMS trials with no visual stimulation, the participants were asked to press freely either the left or right arrow key during the response interval.

Because it was unpredictable and counterbalanced as to whether visual stimuli or TMS would be applied, the participants had to attend to the cued direction in both conditions. Therefore, in TMS trials, either the contralateral or ipsilateral right hemisphere could be perturbed, depending on the attended direction (Fig. 1b). Stimulus delivery was controlled using Psychtoolbox-3^73–75^.

### TMS and EEG recordings

During the spatial attention task, biphasic pulses were applied using a figure-of-eight coil (Double 70 mm Alpha coil; Magstim, UK) connected to the TMS unit (Magstim Super Rapid; Magstim, UK). The TMS target site was located at the upper right V1/V2 according to the calcarine sulcus determined on each individual subject’s MRI (mean Talairach coordinates ± SD: 11, −95 ± 3, 5), and the TMS coil position was near to the O2 electrode for all participants. This TMS location approximately corresponds to the grand averaged alpha power modulated area (see Fig. 1c). The TMS coil and head position were continuously monitored using Brainsight TMS (Rogue Research Inc., Canada), and kept within 5 mm of the initial position. For the sham stimulation, the coil was rotated 90° around the handle axis and spaced from the head using a 3 cm plastic cube^76^. Thus, the participant received some sensation of vibration caused by the TMS click, without receiving direct cortical stimulation. Additionally, the click sound was attenuated by the participant wearing earplugs with air tubes and the delivery of white masking noise in all conditions. The stimulation intensity achieving a 95% active motor threshold (MT) in the right first dorsal interosseous muscle was individually adjusted according to the distance between the TMS coil and targeted visual cortex^77^. This resulted in a stimulation intensity on the visual cortex of 70.5 ± 8.5% maximal stimulator output. We also confirmed that no one perceived any phosphenes.

EEG (left earlobe reference; ground AFZ) signals were recorded from 63 scalp sites using sintered Ag/AgCl TMS-compatible electrodes mounted on a 10/10 EasyCap system (EASYCAP GmbH, Germany). Horizontal and vertical electrooculography (EOG) signals were continuously recorded. The electrode impedance was kept below 10 kΩ. The EEG and EOG signals were amplified and recorded by a Brain Amp MR + (Brain Products GmbH, Germany) system with a sampling rate of 5 kHz. The electrode lead wires were arranged orthogonal to the TMS coil handle direction, to reduce TMS-induced artifacts^63^.

### TMS and ocular artifact rejections

EEG data were analyzed using in-house developed scripts written in MATLAB (MathWorks, USA) and FieldTrip^78^. The data were first segmented into 5 s epochs (3.5 s pre-stimulation and 1.5 s post-stimulation), and then the epoched data were re-referenced offline to the average of the right and left earlobe signals. TMS and ocular artifacts were rejected using the following steps. First, the data samples were temporally smoothed using linear interpolation (two samples preceding and about 21 samples following the TMS onset, i.e., 4.3 ± 0.08 ms) to remove excessive TMS artifacts. Second, epochs contaminated with eye movements and blinks in the interval between −1.3 and 1.1 s were discarded according to the following criteria: horizontal EOG signals exceeding ~50 μV, which approximately corresponded to the stimulus eccentricity (6° visual angle), and vertical EOG exceeding ~100 μV. Third, the exponential decay artifacts due to TMS were attenuated using independent component analysis (ICA) based on the method proposed by Korhonen and colleagues^64^ (see also http://www.fieldtriptoolbox.org/tutorial/tms-eeg for a more practical application). ICs were excluded according to mean z-score values greater than 1.65 between 0 and 50 ms, with the topography of the mixing matrix being confined within the stimulated region. For ocular artifacts, ICs correlating with the EOG (*r* > 0.2, *p* < 0.05) and showing the typical topographical structure of saccadic eye movements and blinks were also excluded. Finally, temporal smoothing linear interpolation was again applied until 10 ms, to remove residual artifact. In addition, we investigated to what extent such interpolation distorts the phase estimate (see Supplementary figure 1). The results suggest that the phase is slightly distorted at higher frequencies, with this being common to all conditions of the stimulation frequency and stimulation site. After discarding epochs contaminated with ocular artifacts, the numbers of trials for real and sham TMS conditions were 119.8 ± 15.5 and 124.3 ± 12.7, respectively. Four participants for whom more than 45% of trials were lost were excluded from further analysis. The artifact-removed EEG measures were then down-sampled to 500 Hz. Current source density (CSD) transformation^79,80^ was applied to localize the activity and attenuate the effects of volume conduction.

### Attentional alpha power modulation

The instantaneous amplitude and phase were obtained using a wavelet transform with a center frequency *f*, time *t*, and standard deviation *σ*_*f*_ = *4 f/m* and *σ*_*t*_ = *m/2πf* ^81^. The constant *m* was set to 3. To determine the pair of electrodes showing the most prominent alpha-power modulated by attention, the alpha modulation index (AMI) for each electrode was computed according to the following: *AMI* = *α*_*left cue*_ *− α*_*right cue*_, where *α*_*left cue*_ and *α*_*right cue*_ refer to the mean alpha power computed from the instantaneous amplitude for the left and right cued trials per electrode. Although the source of alpha power modulation appearing under a visual spatial attention task should be common across individuals, the distribution of the alpha power modulation observed on the scalp differed because of individual differences in head structure. Topographic representations from three representative participants are shown in Fig. 1c. For each participant, the most positive electrode in the left hemisphere and the most negative electrode in the right hemisphere at the time when the AMI was topographically maximized between −1 and −0.1 s were chosen. The average TFRs across participants for the individually selected electrodes are shown in Fig. 1d.

In the next scenario, we investigated how cortical excitability and connectivity changed with the magnitude of the preceding alpha power modulation on a trial by trial basis. Using selected electrodes, the alpha lateralization index was computed as *ALI* = *α*_*ipsilateral electrode*_ − *α*_*contralateral electrode*_, which represents the contrast in alpha power between the ipsilateral and contralateral electrode in respect to the attention side. Then, the 50% of trials with the highest ALI were assigned to “high-ALI” trials, while the other half of trials were assigned to “low-ALI” trials. This meant that the alpha power in the TMS target area (right V1/V2) was relatively low in the left cue trials and high in the right cue trials in the high-ALI trials, while this contrast was marginal in the low-ALI trials.

### TMS evoked potential (TEP)

To investigate the local cortical excitability, we made particular note of an initial cortical reaction in TEP. The preprocessed EEG epochs were bandpass filtered from 3 to 45 Hz using a fourth order Butterworth filter and averaged for each condition. The peak TEP components in the interval 0–0.2 s were identified.

### Inter-area phase synchronization

The effective connectivities between O2 and all other electrodes were assessed using the time-delayed phase locking value (tdPLV) to determine the relationship between sending and receiving areas:

$${\rm{t}}{\rm{d}}PL{V}_{m}=|\frac{1}{N}\mathop{\sum }\limits_{n=1}^{N}\,\exp (i({\varphi }_{O2,n}(f,{t}_{ref})-{\varphi }_{m,n}(f,t)))|,$$

where *φ*_O2_ and *φ*_*m*_ are the instantaneous phase of frequency *f* at electrode O2 and electrode *m* (all the other electrodes). The phase was obtained with its power using the wavelet transform described above in “*Attentional alpha-power modulation*”. *N* denotes the number of trials, *n* is the trial index, and the reference time was set to *t*_*ref*_ = 0, which was the onset timing of the TMS. Thus, tdPLV evaluates the consistency of the phase difference between electrode O2 (sending) at the TMS onset and the other electrodes (receiving) with a given lag-time. It should be noted that if the lag-time is >0, tdPLV provides volume-conduction-free connectivity.

### Statistics

The TEP and tdPLV observed in the contralateral and ipsilateral conditions were statistically evaluated. Note that these were obtained by subtracting sham conditions to reduce the osteoconductive auditory component. For the initial cortical reaction to the TMS perturbation, the P20 component (average of five samples) of the O2 electrode was statistically assessed using a two-way ANOVA with attention-hemisphere (ipsilateral and contralateral) and ALI (low ALI and high ALI) as fixed factors.

For tdPLV, a cluster-based permutation test^82^ was used to verify whether visual spatial attention changed the degree of signal transmission from early visual cortex to other cortical areas. A cluster-based permutation test was used to determine whether the observed difference in the cluster statistics between conditions was large enough to reject the null hypothesis, according to the following procedures. First, all elements (i.e., 63 electrodes, 3–45 Hz, 0–0.5 s) in the tdPLV matrices between the left and right attention conditions in each low-ALI and high-ALI set were compared using two-tailed paired *t*-tests. Then, contiguous negative and positive clusters in the matrices were identified according to an uncorrected *p*-value threshold of <0.05, and the sums of the *t*-values in clusters were calculated as cluster statistics. Second, to obtain the null distribution of the test cluster statistics, the maximal cluster from matrices of randomly permuted two-condition labels within participants was identified, with 2000 iterations being used. Finally, using the 97.5 percentile of the null distribution as the level of statistical significance, significant clusters of observed data were identified. This allowed us to obtain statistically significant clusters across delay time points, frequencies, and electrodes. In other words, the results show significant connections between O2 and other electrodes in respect to delay time and frequency.

## Supplementary information

Supplementary information.

## Data Availability

The datasets generated during and/or analyzed during the current study are available from the corresponding author on reasonable request.
