# Supplementary material for: Probing dynamical cortical gating of attention with concurrent TMS-EEG
Source: Sci Rep. 2020 Mar 18;10:4959. doi: 10.1038/s41598-020-61590-2 (PMC7080792; doi:10.1038/s41598-020-61590-2)
Supplement: Supplementary file 1 — Supplementary information. [file 41598_2020_61590_MOESM1_ESM.docx]

**Probing dynamical cortical gating of attention with concurrent TMS-EEG**

Yuka O. Okazaki^a, b, c^, Yuji Mizuno^a, d^, Keiichi Kitajo^a, b, c,^ *

^a^CBS-TOYOTA Collaboration Center, RIKEN Center for Brain Science, 351-0198, Japan

^b^Division of Neural Dynamics, Department of System Neuroscience, National Institute for Physiological Sciences, National Institutes of Natural Sciences, Okazaki, Aichi, 444-8585, Japan

^c^Department of Physiological Sciences, School of Life Science, The Graduate University for Advanced Studies (SOKENDAI), Okazaki, 444-8585, Japan.

^d^Research Fellow of Japan Society for the Promotion of Science (JSPS), Kojimachi, Chiyoda-ku, Tokyo, Japan

* Corresponding author: Division of Neural Dynamics, Department of System Neuroscience, National Institute for Physiological Sciences, National Institutes of Natural Sciences, 38 Nishigonaka, Myodaiji, Okazaki, Aichi, 444-8585, Japan

*E-mail*: [kkitajo@nips.ac.jp](mailto:kkitajo@nips.ac.jp)


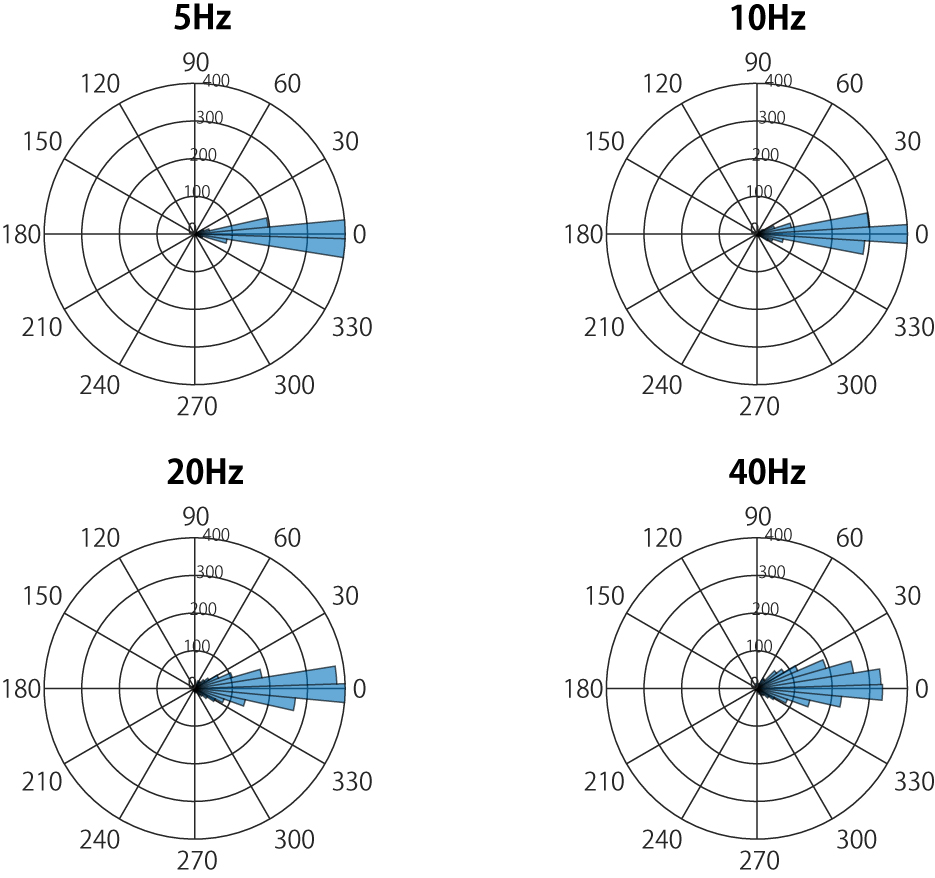


**Supplementary figure 1: Angular histogram of phase difference between original and interpolated data.** For all participants, the original data (epoched from -3.5 to 1.5) from real TMS trials was linearly interpolated from 10 milliseconds, i.e. -1 to -0.99 seconds. Next, the phase difference between the original data and the interpolation data processed in the following procedure was evaluated: (i) Downsample from 5000 to 500; (ii) obtain the phase from the wavelet transform; (iii) calculate the phase angle difference at onset of interpolation. If there is no distortion in the phase of the interpolation data, the phase difference should be zero. Each panel displays an angular histogram of phase differences at frequencies of 5 Hz, 10 Hz, 20 Hz, and 40 Hz. The level of phase difference clustering that can be evaluated as the length of the average phase difference vector in polar space as: $\left| 1/{N\sum_{k}^{N} \exp\left( \varphi_{\mathrm{org}}\left( f \right)-\varphi_{\mathrm{int}}\left( f \right) \right)} \right|$, where 𝜑_org_ and 𝜑_int_ is the phase of frequency f from original and interpolated data, respectively. N is the number of trials from all participants. The measures for phase clustering were 0.98, 0.96, 0.85, and 0.86, respectively (range from 0 to 1).
